# The prognostic effects of tumor infiltrating regulatory T cells and myeloid derived suppressor cells assessed by multicolor flow cytometry in gastric cancer patients

**DOI:** 10.18632/oncotarget.6958

**Published:** 2016-01-20

**Authors:** Han Sol Choi, Sang Yun Ha, Hye-Mi Kim, Soo Min Ahn, Myung-Soo Kang, Kyoung-Mee Kim, Min Gew Choi, Joon Ho Lee, Tae Sung Sohn, Jae Moon Bae, Sung Kim, Eun-Suk Kang

**Affiliations:** ^1^ Samsung Biomedical Research Institute, Samsung Medical Center, Sungkyunkwan University School of Medicine, Seoul, Korea; ^2^ Department of Laboratory Medicine and Genetics, Samsung Medical Center, Sungkyunkwan University School of Medicine, Seoul, Korea; ^3^ Department of Pathology and Translational Genomics, Samsung Medical Center, Sungkyunkwan University School of Medicine, Seoul, Korea; ^4^ Samsung Advanced Institute for Health Sciences and Technology (SAIHST), Samsung Medical Center, Sungkyunkwan University School of Medicine, Seoul, Korea; ^5^ Samsung Biomedical Research Institute (SBRI), Center for Future Sciences, Samsung Medical Center, Sungkyunkwan University School of Medicine, Seoul, Korea; ^6^ Department of Surgery, Samsung Medical Center, Sungkyunkwan University School of Medicine, Seoul, Korea

**Keywords:** regulatory T cells, myeloid derived suppressor cells, prognosis, gastric cancer

## Abstract

The prognostic effects of tumor infiltrating lymphocytes (TILs), especially regulatory T cells (Tregs) and myeloid derived suppressing cells (MDSCs) are inconclusive in gastric cancers. We investigated the frequencies of TILs including CD8+ T cells, CD45+CD4+CD25± FOXP3+ Tregs, CD45+CD11b+ CD14+ HLA−DR− MDSCs in 28 gastric cancer tissues by using multicolor flow cytometry. In gastric cancer tissue, the percentage of Tregs among the CD4+ T cell subset was substantially increased compared to that of Tregs among peripheral blood CD4+ T cells from the controls. High frequency of CD8+ T cells among CD3+ T cells correlated with increased overall survival (OS) (*p* = 0.005). High frequency of Tregs among CD4+ T cells correlated with increased OS (*p* < 0.001), and disease-free survival (DFS) (*p* = 0.039) and was an independent prognostic factor in OS (Hazard ratio: 0.047; 95% confidence interval, 0.006-0.372; *p* = 0.004). High frequency of MDSCs among total examined cells correlated with decreased OS (*p* = 0.027) and was an independent prognostic factor in OS (Hazard ratio 8.601; 95% confidence interval, 1.240-59.678; *p* = 0.029). We have demonstrated that high levels of Tregs among tumor-infiltrating CD4+ T cells were favorable, but an increased proportion of MDSCs was an adverse independent prognostic factor in gastric cancer. Our results may provide important insights for future immunotherapy in gastric cancer.

## INTRODUCTION

Gastric cancer (GC) is the fourth most commonly diagnosed cancer and the second most common cause of cancer-related deaths worldwide [1, 2]. Despite efforts to introduce new treatment modalities such as surgery combined with chemotherapy or chemo-radiotherapy, the control of GC at an advanced stage remains difficult [3]. Considering the high mortality rate of GC in advanced stage or at recurrence, new therapeutic strategies are urgently needed.

Mechanisms underlying the dynamic interplay between immune cells and tumor progression have been studied during past decades. The accumulated data indicate that the outcome of an immune response toward a tumor is largely determined by the type of immune response elicited [4]. Earlier studies have shown survival benefit of tumor infiltrating lymphocytes (TILs) [5–8], and this suggests that TILs are effective on suppressing tumor progression. Thereafter, subgrouping of CD3+ T cells into cytotoxic T cells and regulatory T cells (Tregs) have made further understanding of relationship between TILs and cancer progression. Infiltration of CD8+ cytotoxic T cells into tumors has been reported as a favorable prognostic factor in many cancers including GCs [9–17]. Tregs regulate immune reactivity including tumor-specific immune responses [18]. Tregs were initially characterized by the CD4+CD25+ phenotype, but the most specific cell marker of Tregs identified to date is the nuclear transcription factor known as Forkhead box protein P3 (FOXP3) [19–23]. Many studies have shown that a high density of Tregs, which usually evaluated by quantification of FOXP3+ T cells by using immunohistochemistry (IHC), was associated with a poor outcome, and these results were explained on the basis of the suppressive effects of Tregs on antitumor cytotoxic T cells [24–26]. However, many controversial results have been reported [24, 27]. Moreover, the controversial prognostic significance of Tregs may differ depending on the specific tumor types. According to a recent meta-analyses, the prognostic impacts of Treg infiltration is not consistent [27]. For example, Treg is a favorable prognostic factor in colorectal carcinoma while it is an adverse prognostic factor in hepatocellular carcinoma [24]. In GC, the prognostic significance of Tregs has been controversial. Many studies reported Tregs as adverse prognostic factor in GC [25, 27–29], while several opposite results suggesting Tregs as a favorable prognostic factor have been reported so far [18, 30, 31].

Myeloid-derived suppressor cells (MDSCs) are a heterogeneous population of immature myeloid cells and are involved in inhibiting both innate and adaptive immune responses [32, 33]. In cancer biology, MDSCs have been shown to potently inhibit antitumor immunity *in vivo* through diverse mechanisms [32, 33]. MDSCs have been generally defined as a CD11b+ CD33+ CD14+ HLA− DR− myeloid cell population in human cancer patients [33]. In GC, two previous studies have shown that the numbers of MDSCs are increased in the blood of cancer patients compared with healthy individuals, and this increase was associated with adverse clinical outcomes [34, 35]. However, the clinical significance of MDSCs in GC tissue remains completely unknown because specific phenotype of MDSCs cannot be evaluated by IHC in cancer tissue.

In this study, we examined the frequencies of tumor infiltrating immune cell subsets by multi-color flow cytometry, which enabled us to define more specific roles of immune cells as well as more objective quantification in GC, and investigated the clinical significance of immune cells, especially Treg and MDSC.

## RESULTS

### Distribution of immune cell subsets in gastric cancer tissue

The frequencies of the various immune cell subsets in the GC tissue are summarized in Table 1. The frequencies of immune cell subsets in PBMCs of 8 healthy individuals measured using same immunophenotyping panels are displayed. In GC tissue samples, CD45+ hematopoietic cells occupied 8.5% (median value, range 0.8–29.4%) of the total counted cells, and lymphocytes and myeloid cells occupied 68.4% and 31.6% of the CD45+ cells, respectively.

**Table 1:** Distribution of lymphocyte subsets in gastric cancer tissue and peripheral blood mononuclear cells of healthy individuals

Immunophenotypes	Subsets	% of subsets (median, range)	
		Gastric cancer tissue (*n* = 28)	Peripheral blood from healthy control (*n* = 8)
CD45+ leukocyte among total cells	Leukocytes	8.45 (0.8-29.4)	100
Lymphocyte (size gating) among CD45+ leukocytes	Lymphocyte	4.15 (0.3-22.0)	19.3 (14.4-28.4)
CD3+CD4+ among CD3+ cells	Helper T cells	56.6 (20.9-83.4)	59.4 (29.1-74.0)
CD4+FOXP3+ among CD4+ cells	Regulatory T cells	12.7 (1.7-37.6)	4.1 (0.5-8.5)
CD3+CD8+ among CD3+ cells	Cytotoxic T cells	42.2 (16.6-79.1)	38.3 (3.1-47.2)
CD56+ among lymphocytes	NK cells	0.1 (0-3.0)	10.3 (1.3-27.1)
CD45+CD11b+CD14+ HLA−DR− among CD45+ leukocytes	MDSCs	2.8 (0.6-13.6)	0.35 (0.1-3.4)

The percentage of Tregs among the CD4+ T cells and MDSCs among CD45+ leukocytes in GC tissue from cancer patients was median 12.7% (range 1.7–37.6%) and median 2.8% (range 0.6–13.6%), respectively. The percentage of Tregs among the CD4+ T cells and MDSCs among CD45+ leukocytes in peripheral blood from healthy donors was median 4.1% (range 0.5–8.5%) and median 0.35% (range 0.1–3.4%), respectively. Unexpectedly, CD56+ NK cells were scarcely observed in the GC tissues (proportion of NK cells among lymphocytes, median 0.1%; range 0–3%).

### The classification according to frequencies of immune cells by two strategies: among total examined cells or their belonging subset of immune cells

The cases were dichotomized into low-density and high-density group by two strategies: (1). Frequencies among total examined cells; (2). Frequencies among their belonging subsets of immune cells. According to the frequency of CD8+ T cells (CD8/Total), Tregs (Treg/Total), and MDSCs (MDSC/Total) among total examined, cases were sub-classified into the low density group (CD8^low^/Total, Treg^low^/Total, MDSC^low^/Total) and the high density groups (CD8^high^/Total, Treg^high^/Total, MDSC^high^/Total) based on the cutoff determined using the X-tile package (CD8/Total, 2.0%; Treg/Total, 0.7%; MDSC/Total, 0.24%). According to the frequency of CD8+ T cells among CD3+ T cells (CD8/CD3), Tregs among CD4+ helper T cells (Treg/CD4), and MDSCs among CD45+ leukocytes (MDSC/CD45), cases were dichotomized into the low density (CD8^low^/CD3, Treg^low^/CD4, MDSC^low^/CD45) and the high density groups (CD8^high^/CD3, Treg^high^/CD4, MDSC^high^/CD45) based on the cutoff levels determined with X-tile package as follows: CD8/CD3, 30%; Treg/CD4, 9%; MDSC/CD45, 2.2%. The associations of clinicopathologic features and frequencies of immune cells among total examined cells and their belonging subsets of immune cells were summarized in Table 2 and 3.

**Table 2:** Clinicopathologic features according to the proportions of CD8+ T cells, regulatoryT cells, and myeloid derived suppressor cells among total cells

		Total*n* (%)	CD8/Total		*p*	Treg/Total		*p*	MDSC/Total		*p*
			Low *n* (%)	High *n* (%)		Low *n* (%)	High *n* (%)		Low *n* (%)	High *n* (%)	
Age	≥ 50	8 (28.6)	3 (17.6)	5 (45.5)	0.200	1 (11.1)	7 (36.8)	0.214	**7 (46.7)**	**1 (7.7)**	**0.038**
	< 50	20 (71.4)	14 (82.4)	6 (54.5)		8 (88.9)	12 (63.2)		**8 (53.3)**	**12 (92.3)**	
Gender	Female	10 (35.7)	4 (23.5)	6 (54.5)	0.125	2 (22.2)	8 (42.1)	0.417	7 (46.7)	3 (23.1)	0.254
	Male	18 (64.3)	13 (76.5)	5 (45.5)		7 (77.8)	11 (57.9)		8 (53.3)	10 (76.9)	
Size	≥ 8 cm	13 (46.4)	8 (47.1)	5 (45.5)	0.934^1^	5 (55.6)	8 (42.10	0.689	7 (46.7)	6 (46.2)	1
	< 8 cm	15 (53.6)	9 (52.9)	6 (54.5)		4 (44.4)	11 (57.9)		8 (53.3)	7 (53.8)	
Differentiation	W/D, M/D	10 (35.7)	8 (47.1)	2 (18.2)	0.226	5 (55.6)	5 (26.3)	0.210	6 (40.0)	4 (30.8)	0.705
	P/D	18 (64.3)	9 (52.9)	9 (81.8)		4 (44.4)	14 (73.7)		9 (60.0)	9 (69.2)	
Lauren	Intestinal	15 (53.6)	10 (58.8)	5 (45.5)	0.488^1^	7 (77.8)	8 (42.1)	0.114	9 (60.0)	6 (46.2)	0.705
	Diffuse	13 (46.4)	7 (41.2)	6 (54.5)		2 (22.2)	11 (57.9)		6 (40.0)	7 (53.8)	
T stage	2	1 (3.6)	0 (0)	1 (9.1)	0.642	0 (0)	1 (5.3)	0.194	1 (6.7)	0 (0)	1
	3	9 (32.1)	6 (35.3)	3 (27.3)		5 (55.6)	4 (21.1)		5 (33.3)	4 (30.8)	
	4	18 (64.3)	11 (64.7)	7 (63.6)		4 (44.4)	14 (73.7)		9 (60.0)	9 (69.2)	
N stage	0	2 (7.1)	0 (0)	2 (18.2)	0.263^2^	0 (0)	2 (10.5)	0.456^2^	1 (6.7)	1 (7.7)	0.195^2^
	1	4 (14.3)	2 (11.8)	1 (9.1)		0 (0)	3 (15.8)		0 (0)	3 (23.1)	
	2	7 (25.0)	5 (29.4)	3 (27.3)		4 (44.4)	4 (21.1)		4 (26.7)	4 (30.8)	
	3	15 (53.6)	10 (58.8)	5 (45.5)		5 (55.6)	10 (52.6)		10 (66.7)	5 (38.5)	
TNM stage	IIA, IIB	3 (10.7)	0 (0)	3 (27.3)	0.781^2^	0 (0)	3 (15.8)	0.846^2^	**1 (6.7)**	**2 (15.4)**	**0.033^2^**
	IIIA	4 (14.3)	4 (23.5)	0 (0)		1 (11.1)	3 (15.8)		**1 (6.7)**	**3 (23.1)**	
	IIIB	7 (25.0)	6 (35.3)	1 (9.1)		6 (66.7)	1 (5.3)		**3 (20.0)**	**4 (30.8)**	
	IIIC	10 (35.7)	6 (35.3)	4 (36.4)		1 (11.1)	9 (47.4)		**6 (40.0)**	**4 (30.8)**	
	IV	4 (14.3)	1 (5.9)	3 (27.3)		1 (11.1)	3 (15.8)		**4 (26.7)**	**0 (0)**	
Recurrence	No	15 (53.6)	7 (41.2)	8 (72.7)	0.102^1^	3 (33.3)	12 (63.2)	0.228	10 (66.7)	5 (38.5)	0.136^1^
	Yes	13 (46.4)	10 (58.8)	3 (27.3)		6 (66.7)	7 (36.8)		5 (33.3)	8 (61.5)	
Death	No	19 (67.9)	10 (58.8)	9 (81.8)	0.249	4 (44.4)	15 (78.9)	0.097	**13 (86.7)**	**6 (46.2)**	**0.042**
	Yes	9 (32.1)	7 (41.2)	2 (18.2)		5 (55.6)	4 (21.1)		**2 (13.3)**	**7 (53.8)**	

1 By Chi-Square test

2 By Cochran Armitage test, otherwise by Fisher's exact test.

Bold letter represent statistics having significance with *p* < 0.05.

**Table 3:** Clinicopathologic features according to the proportions of CD8+ T cells among CD3+T cells, regulatory T cells among CD4+ helper T cells, and myeloid derived suppressor cells among CD45+ leukocytes

		Total	CD8/CD3		*p*	Treg/CD4		*p*	MDSC/CD45		*p*
			Low *n* (%)	High *n* (%)		Low *n* (%)	High *n* (%)		Low *n* (%)	High *n* (%)	
Age	≥ 50	8 (28.6)	0 (0)	8 (33.3)	0.295	2 (25.0)	6 (30.0)	1.000	3 (37.5)	5 (25.0)	0.651
	< 50	20 (71.4)	4 (100)	16 (66.7)		6 (75.0)	14 (70.0)		5 (62.5)	15 (75.0)	
Gender	Female	10 (35.7)	0 (0)	10 (41.7)	0.265	2 (25.0)	8 (40.0)	0.669	4 (50.0)	6 (30.0)	0.400
	Male	18 (64.3)	4 (100)	14 (58.3)		6 (75.0)	12 (60.0)		4 (50.0)	14 (70.0)	
Size	≥ 8 cm	13 (46.4)	0 (0)	13 (54.2)	0.102	4 (50.0)	9 (45.0)	1.000	2 (25.0)	11 (55.0)	0.221
	< 8 cm	15 (53.6)	4 (100)	11 (45.8)		4 (50.0)	11 (55.0)		6 (75.0)	9 (45.0)	
Differentiation	W/D, M/D	10 (35.7)	2 (50.0)	8 (33.3)	0.601	2 (25.0)	8 (40.0)	0.669	3 (37.5)	7 (35.0)	1.000
	P/D	18 (64.3)	2 (50.0)	16 (66.7)		6 (75.0)	12 (60.0)		5 (62.5)	13 (65.0)	
Lauren	Intestinal	15 (53.6)	3 (75.0)	12 (50.0)	0.600	5 (62.5)	10 (50.0)	0.686	4 (50.0)	11 (55.0)	1.000
	Diffuse	13 (46.4)	1 (25.0)	12 (50.0)		3 (37.5)	10 (50.0)		4 (50.0)	9 (45.0)	
T stage	2	1 (3.6)	0 (0)	1 (4.2)	0.166^1^	0 (0)	1 (5.0)	0.915^1^	0 (0)	1 (5.0)	715^1^
	3	1, 9	3 (75.0)	6 (25.0)		3 (37.5)	6 (30.0)		4 (50.0)	5 (25.0)	
	4	18 (64.3)	1 (25.0)	17 (70.8)		5 (62.5)	13 (65.0)		4 (50.0)	14 (70.0)	
N stage	0	2 (7.1)	**1 (25.0)**	**1 (4.2)**	**0.023^1^**	1 (12.5)	1 (5.0)	0.1861	1 (12.5)	1 (5.0)	0.833^1^
	1	4 (14.3)	**1 (25.0)**	**2 (8.3)**		1 (12.5)	2 (10.0)		0 (0)	3 (15.0)	
	2	7 (25.0)	**1 (25.0)**	**7 (29.2)**		3 (37.5)	5 (25.0)		2 (25.0)	6 (30.0)	
	3	15 (53.6)	**1 (25.0)**	**14 (58.3)**		3 (37.5)	12 (60.0)		5 (62.5)	10 (50.0)	
TNM stage	IIA, IIB	3 (10.7)	0 (0)	3 (12.5)	0.604^1^	0 (0)	3 (15.0)	0.547^1^	0 (0)	3 (15.0)	0.123^1^
	IIIA	4 (14.3)	2 (50.0)	2 (8.3)		2 (25.0)	2 (10.0)		1 (12.5)	3 (15.0)	
	IIIB	7 (25.0)	1 (25.0)	6 (25.0)		2 (25.0)	5 (25.0)		2 (25.0)	5 (25.0)	
	IIIC	10 (35.7)	0 (0)	10 (41.7)		2 (25.0)	8 (40.0)		2 (25.0)	8 (40.0)	
	IV	4 (14.3)	1 (25.0)	3 (12.5)		2 (25.0)	2 (10.0)		3 (37.5)	1 (5.0)	
Recurrence	No	15 (53.6)	1 (25.0)	14 (58.3)	0.311	2 (25.0)	13 (65.0)	0.096	2 (25.0)	13 (65.0)	0.096
	Yes	13 (46.4)	3 (75.0)	10 (41.7)		6 (75.0)	7 (35.0)		6 (75.0)	7 (35.0)	
Death	No	19 (67.9)	1 (25.0)	18 (75.0)	0.084	**2 (25.0)**	**17 (85.0)**	**0.005**	4 (50.0)	15 (75.0)	0.371
	Yes	9 (32.1)	3 (75.0)	6 (25.0)		**6 (75.0)**	**3 (15.0)**		4 (50.0)	5 (25.0)	

1 By Cochran Armitage test, otherwise by Fisher's exact test.

Bold letter represent statistics having significance with *p* < 0.05.

### High frequency of CD8+ T cells among CD3+ T cells correlates with increased overall survival

**Figure 1:**
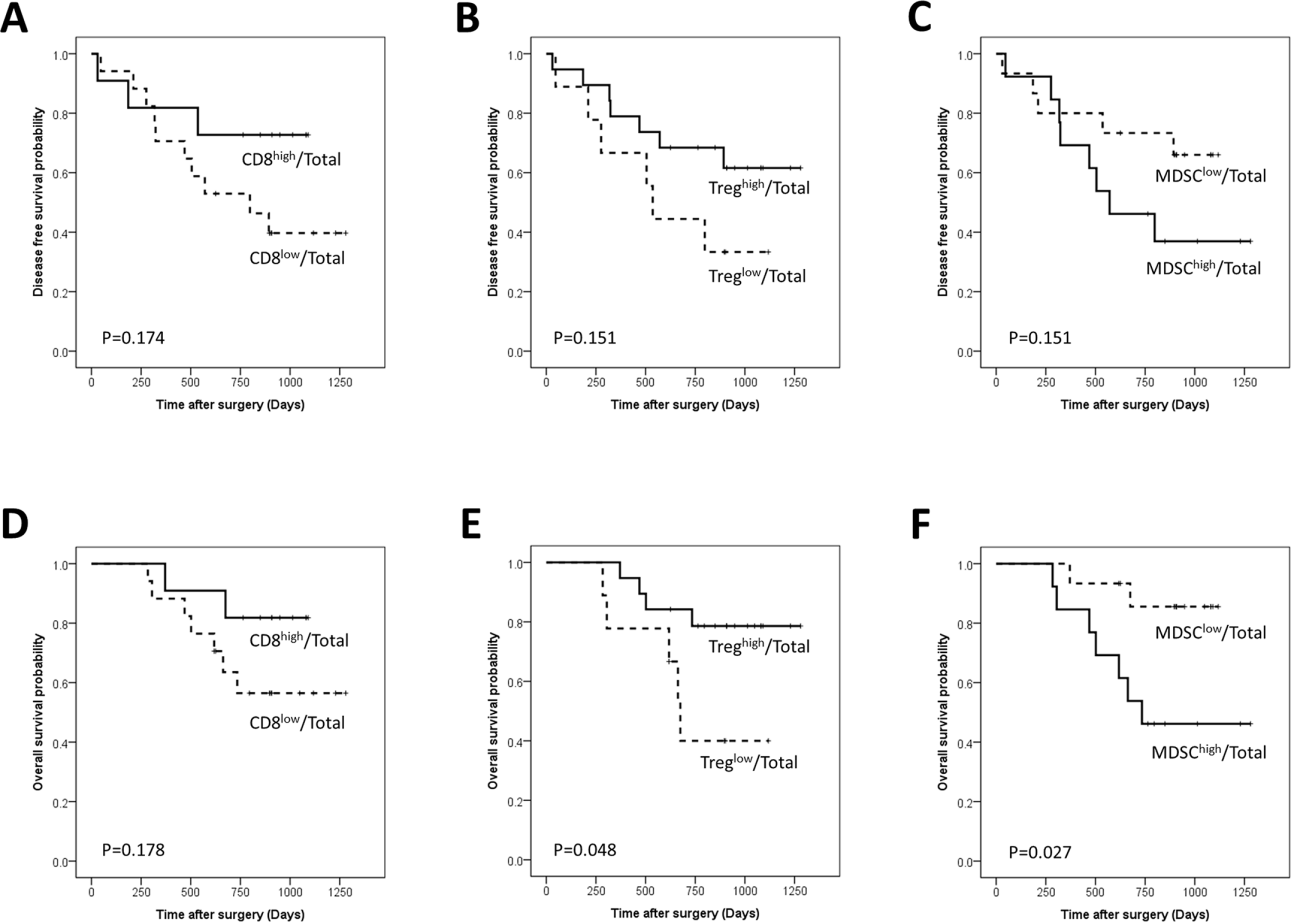
Disease-free and overall survival curves of gastric cancer patients according to the proportions of CD8+ T cells (A, D), regulatory T cells (Tregs) (B, E) and Myeloid derived suppressor cells (MDSCs) (C, F) among total examined cells

**Figure 2:**
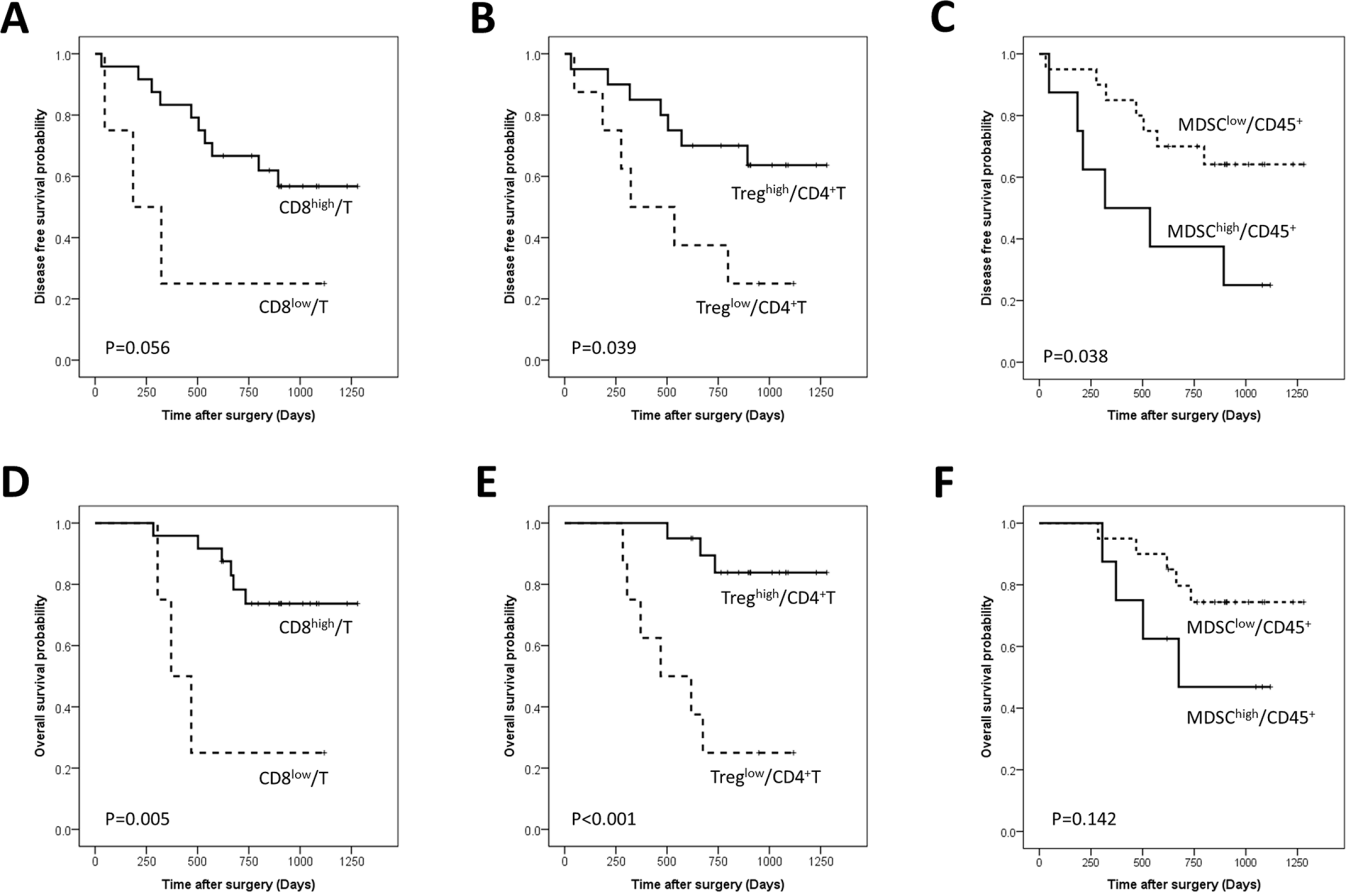
Disease-free and overall survival curves of gastric cancer patients according to the proportions of CD8+ T cells among CD3+ T cells (A, D), regulatory T cells (Tregs) among CD4+ T cells (B, E), and Myeloid derived suppressor cells (MDSCs) among CD45+ leukocytes (C, F)

The CD8^high^/Total patient subgroup was associated with longer DFS and OS compared with the CD8^low^/Total patient subgroup, but the tendency did not reach statistical significance (Figure 1A, 1D). The proportion of the CD8^high^/CD3 group was tended to increase according to the advancing N stage (*p* = 0.023) (Table 3). The CD8^high^/CD3 group showed longer OS (*p* = 0.005) and a tendency towards longer DFS than the CD8^low^/CD3 group (*p* = 0.056) (Figure 2A, 2D).

### High frequency of Tregs among CD4+ T cells correlates with increased overall survival and is an independent prognostic factor in overall survival

The Treg^high^/Total group showed significantly longer OS (*p* = 0.048) and a tendency of longer DFS compared to the Treg^low^/Total (Figure 1B, 1E). The Treg^high^/CD4 group showed longer OS (*p* < 0.001)) and DFS (*p* = 0.039) than the Treg^low^/CD4 group (Figure 2B, 2E) and remained a significant predictor of OS in the multivariate analysis (HR: 0.065; 95% CI, 0.009–0.491; *p* = 0.008) (Table 4).

**Table 4:** The results of univariate and multivariate analyses

		Disease free survival					
		Univariate			Multivariate		
		HR	95% CI	*p* value	HR	95% CI	*p* value
Age	≥ 50 vs < 50	3.153	0.696-14.295	0.136	1.767	0.224-13.974	0.589
Gender	Male vs female	1.504	0.462-4.895	0.498			
Size	≥ 8 cm vs < 8 cm	1.870	0.608-5.751	0.275	2.059	0.605-7.013	0.248
Lauren	Diffuse or mixed vsintestinal	1.302	0.437-3.879	0.635			
Differentiation	P/D vs W/D or M/D	0.767	0.250-2.349	0.642			
TNM stage	IIIB-IV vs II, IIIA	2.318	0.513-10.472	0.275	2.63	0.529-13.083	0.238
CD8/Total	High vs low	0.419	0.115-1.527	0.188	0.933	0.173-5.049	0.936
Treg/Total	High vs low	0.458	0.153-1.367	0.162	0.481	0.124-1.866	0.290
MDSC/Total	High vs low	2.246	0.724-6.695	0.161	2.171	0.573-8.222	0.254
Age	≥ 50 vs < 50	3.153	0.696-14.295	0.136	**8.987**	**1.275-63.359**	**0.028**
Gender	Male vs female	1.504	0.462-4.895	0.498			
Size	≥ 8 cm vs < 8 cm	1.870	0.608-5.751	0.275	1.659	0.299-9.208	0.563
Lauren	Diffuse or mixed vsintestinal	1.302	0.437-3.879	0.635			
Differentiation	P/D vs W/D or M/D	0.767	0.250-2.349	0.642			
TNM stage	IIIB-IV vs II, IIIA	2.318	0.513-10.472	0.275	7.271	0.788-67.136	0.080
CD8/CD3	High vs low	0.300	0.081-1.110	0.071	1.535	0.074-31.937	0.782
Treg/CD4	High vs low	**0.331**	**0.110-0.994**	**0.049**	0.259	0.042-1.618	0.149
MDSC/CD45	High vs low	**3.024**	**1.010-9.055**	**0.048**	5.151	0.938-28.292	0.059

		Overall survival					
		Univariate			Multivariate		
		HR	95% CI	*p* value	HR	95% CI	*p* value
Age	≥ 50 vs < 50	3.898	0.487-31.205	0.200	0.976	0.076-12.597	0.985
Gender	Male vs female	1.175	0.293-4.707	0.820			
Size	≥ 8 cm vs < 8 cm	1.915	0.478-7.670	0.359			
Lauren	Diffuse or mixed vs intestinal	1.267	0.340-4.722	0.724			
Differentiation	P/D vs W/D or M/D	0.958	0.239-3.846	0.952			
TNM stage	IIIB-IV vs II, IIIA	3.119	0.389-24.990	0.284	3.603	0.392-33.125	0.257
CD8/Total	High vs low	0.354	0.073-1.713	0.197	1.087	0.164-7.199	0.931
Treg/Total	High vs low	0.284	0.075-1.070	0.063	0.226	0.043-1.181	0.078
MDSC/Total	High vs low	**4.955**	**1.027-23.897**	**0.046**	**8.601**	**1.240-59.678**	**0.029**
Age	≥ 50 vs < 50	3.898	0.487-31.205	0.200	21.658	0.941-498.647	0.055
Gender	Male vs female	1.175	0.293-4.707	0.820			
Size	≥ 8 cm vs < 8 cm	1.915	0.478-7.670	0.359			
Lauren	Diffuse or mixed vsintestinal	1.267	0.340-4.722	0.724			
Differentiation	P/D vs W/D or M/D	0.958	0.239-3.846	0.952			
TNM stage	IIIB-IV vs II, IIIA	3.119	0.389-24.990	0.284	24.216	1.131-518.706	0.042
CD8/CD3	High vs low	**0.164**	**0.039-0.684**	**0.013**	0.786	0.021-29.689	0.897
Treg/CD4	High vs low	**0.113**	**0.028-0.460**	**0.002**	**0.047**	**0.006-0.372**	**0.004**
MDSC/CD45	High vs low	2.598	0.692-9.755	0.157	2.477	0.186-32.892	0.492

### Low frequency of MDSCs among total examined cells correlates with increased overall survival and is an independent prognostic factor in overall survival

The MDSC^low^/Total group was more frequent in old age patients and the proportion of MDSC^high^/Total group decreased as the TNM stage advanced (Table 2). The MDSC^low^/Total group showed a significantly longer OS (*p* = 0.027) and a tendency of longer DFS compared to the MDSC^high^/Total groups (*p* = 0.151) (Figure 1C, 1F). MDSC/Total was an independent prognostic factor (HR 8.601; 95% CI, 1.240–59.678; *p* = 0.029) in OS (Table 4). MDSC^high^/CD45+ group showed better DFS rate (*p* = 0.038) and a tendency of better OS than MDSC^low^/CD45+ group (*p* = 0.142) (Figure 2C, 2F)

## DISCUSSION

In this study, we explored the frequencies of various immune cell subsets in GC tissues by multi-color flow cytometry and evaluated the prognostic value of intratumoral CD8+ cytotoxic T cell, Treg and MDSC frequencies. We found that GC tissues contained significant proportions of immune cells including Tregs and MDSCs and the percentages of Tregs among CD4+ T cells and of MDSCs among myeloid cells were substantially higher when compared with those in peripheral blood from healthy individuals. Moreover, the high proportion of Tregs among CD4+ T cells was an independent prognostic factor for longer OS, whereas the increased proportion of MDSCs among total examined cells was associated with shorter OS.

Infiltration of CD8+ cytotoxic T cells into tumors has been reported as a favorable prognostic factor in many cancers including GCs [10–17]. Here, we report that higher frequencies of CD8+ T cell among tumor-infiltrating CD3+ T cells are associated with longer OS. The prognostic significance of Tregs infiltration remains controversial and seems different depending on the specific tumor type. Many studies have shown that a high density of Tregs in IHC results is associated with a poor outcome, and these results were explained on the basis of the suppressive effects of Tregs on antitumor cytotoxic T cells [24–26]. However, a few recent studies have highlighted the protective effects of Tregs, especially in gastric or colorectal cancers occurring in an inflammatory background caused by microorganisms [9, 14, 28–30]. In this context, Tregs were shown to inhibit the immune reaction of cytotoxic T cells in response to bacteria rather than cancer cells, and, thereby, to suppress cancer progression by controlling inflammation. In GC, the results of several studies on TILs focused on Tregs are controversial [9, 28, 31–33]. This discrepancy could be explained by differences in technical issue; specific FOXP3 antibodies, scoring or statistical process. Moreover, most of the previous studies defined Tregs as FoxP3+ cells by IHC. Unlike previous studies using a single marker to detect a specific type of immune cell by IHC followed by manual quantification of positive cells, we used flow cytometry to analyze the cell populations at the single-cell level. This approach made possible to identify cell lineages or subsets by using multiple monoclonal antibodies at the same time and utilizing objective quantification with an automated system. So, overview of the immune cell population from the cancer tissue was possible and we determined the percentage of immune cells among the total number of examined cells as well as the proportion of each immune cells among their belonging subsets of immune cells.

In this study, we could define tumor infiltrating Tregs in detail as CD45+CD4+CD25± and FOXP3+ cells and found that a higher frequency of Tregs among total examined cells was associated with a longer OS, and this result is in line with several prior reports [9, 33]. Furthermore, we found that the proportion of Tregs among CD4+ T cells was an independent favorable prognostic factor, which could not be evaluated by traditional IHC method. In addition to traditional concept of Tregs suppressing antitumor cytotoxic T cells, several other subsets of Treg have been identified, and direct tumor-killing Tregs activity has been reported [34, 35]. The functional relevance of Treg-mediated tumor killing remains unknown. Further functional studies of Tregs may elucidate their role in the antitumor response and may help to explain the observed associations with the disease prognosis.

MDSCs are generally defined based on the combined expression of several markers and cannot simply be identified by IHC in cancer tissue. Therefore, MDSCs have been rarely studied in cancer tissue. Recently, Sun et al. reported that CD33−HLA−DR− MDSC were increased in the peripheral blood and tumor tissue of colorectal cancer patients compared to the peripheral blood of healthy controls [36]. They also found that an increased frequency of MDSCs was correlated with nodal/distant metastasis and tumor stage. In GC, two previous studies showed increased MDSCs in PBMCs from cancer patients and that MDSC frequency was associated with adverse clinical outcomes [37, 38]. In the present study, we found that high MDSC percentages among total cells were an independent adverse prognostic factor in patients with advanced GC. This result may be explained by the role of MDSCs as inhibitors of antitumor immunity [39–42].

Although this prospective study provides valuable insights on de novo presence and relative quantity of multiple immune cell subsets in GC tissues, current study has limitations as follow: First, it was performed in relatively small number of patients with short follow up period. Second, the cutoffs, which were determined with the highest statistical significance for patient survival in presents study, need to be validated in an independent cohort. Third, the location of the immune cells within the tumor remains unknown whether the immune cells are in the intraepithelial, intratumoral or peritumoral stroma. To improve our understanding of the complex role of immune cells in GC, further studies combining both flow cytometry and IHC on a larger number of cases are necessary.

In conclusion, we have demonstrated that high levels of Tregs among tumor-infiltrating CD4+ T cells were favorable, but an increased proportion of MDSCs was an adverse independent prognostic factor in GC. Our results may provide important insights for further classification of GC based on immunological feature and also for future immunotherapy in GC.

## MATERIALS AND METHODS

### Patients

Tissue samples from 28 patients with GC undergoing curative resection of the primary tumor at the Department of Surgery at the Samsung Medical Center between June 2010 and September 2011 were included in this study (18 males, 10 females; ages ranging from 37 to 88 years). The research protocol was approved by the Institutional Review Board. Written informed consent was received from all patients. Tumor stage and grading were classified according to the 7th edition of the TNM Classification of the International Union Against Cancer [43]. Patients with other malignant diseases in their medical history and patients treated with neoadjuvant chemotherapy were excluded from this study. Six patients had postoperative concurrent chemo-radiation therapy and 13 patients performed postoperative chemotherapy. The median follow-up period was 29 months (range 10–42 months).

Tumor tissues were taken from the center of the cancer immediately after surgical removal and histologic confirmation. For preparation of single cell suspensions, tumor samples were mechanically dissected and washed with PBS. The cell suspension was passed through a sterile 40 μm nylon filter (BD Falcon, Heidelberg, Germany), and single cells were pelleted and suspended in PBS. Data from peripheral blood mononuclear cells from 8 healthy individuals were used as a control.

### Multicolor flow cytometry

Multicolor immunofluorescence and multi-parameter flow cytometric analyses were used to characterize the single-cell suspensions. To distinguish TILs from the tumor cells, initial gating on anti-CD45 (2D1; BD Biosciences, San Jose, CA) positive cells was performed. Afterwards, anti-CD3 (SK7; BD Biosciences, San Jose, CA), anti-CD4 (RPA-T4; eBioscience, San Diego, CA), anti-CD8 (PRA-T8; BD Biosciences, San Jose, CA), anti-CD25 (BC96; eBioscience), and anti-Foxp3 (PCH101; eBioscience) were applied for the identification of the T cell subsets. To detect the NK subset, anti-CD56 (MEM188; eBioscience) and anti-CD16 (CB16; eBioscience) were applied. Finally, to identify MDSCs, anti-HLA-DR (LN3; eBioscience), anti-CD11b (ICRF44; eBioscience), anti-CD14 (61D3; eBioscience) and 7-AAD (eBioscience) for live cell gating were used. The unstained cells and cells stained with relevant isotype antibodies were used as controls. The antibodies listed above are all mouse anti-human monoclonal antibodies.

The cells were incubated with the surface marker antibodies for 30 min at 4°C and washed twice with PBS. To conduct intracellular staining, 1 ml of fixation and permeabilization mixture (eBioscience) was added, and the cells were incubated on ice for an hour. After incubation, the cells were washed twice with PBS. After washing, the cells were mixed with the antibodies and incubated at room temperature for 15 minutes. After the final wash, the cells were resuspended in PBS buffer and analyzed with a FACS Canto II Flow Cytometer (BD Biosciences; FACS DIVA software ver. 6.1.3).

### Data analysis

**Figure 3:**
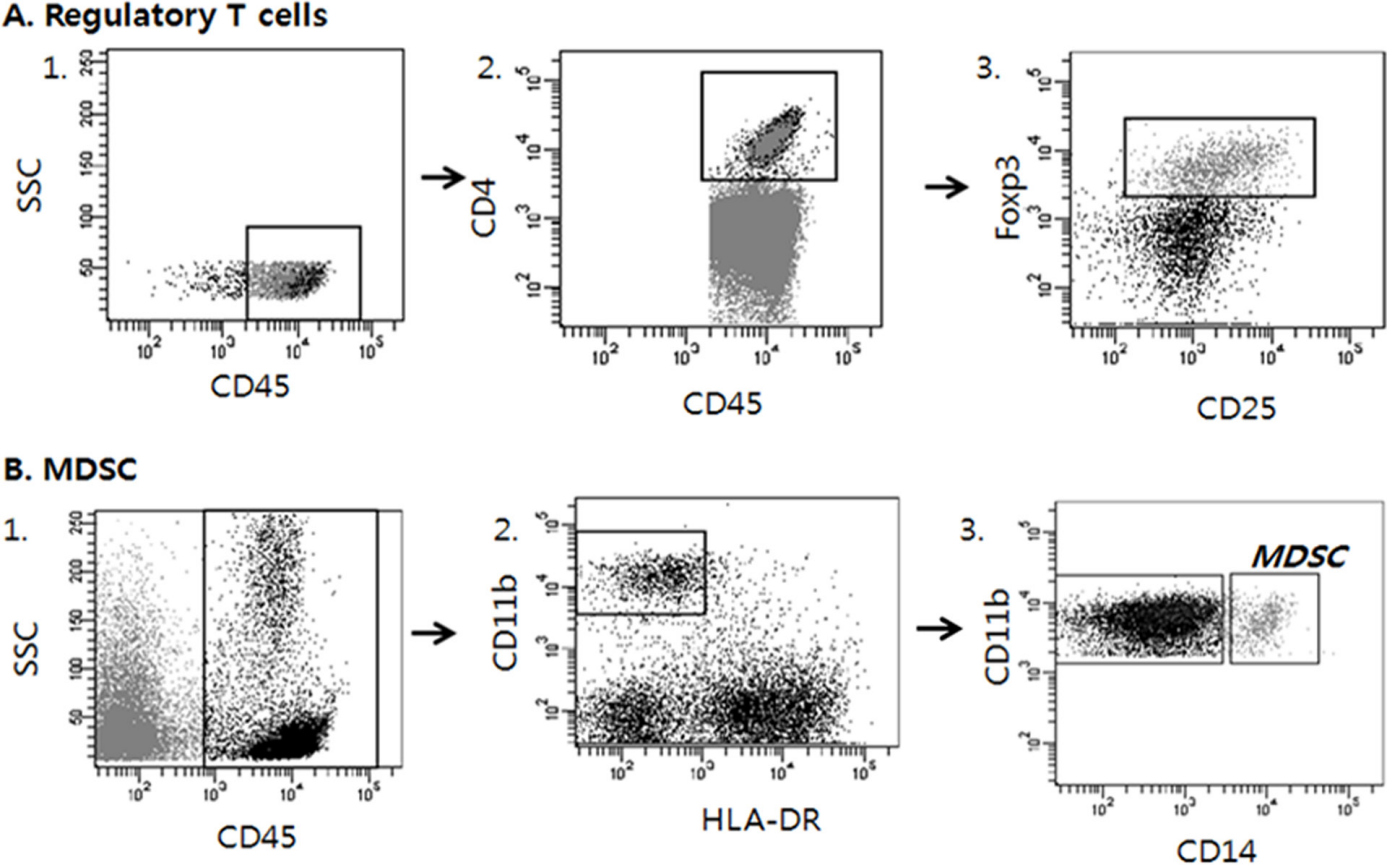
Analysis strategy to detect regulatory T cells (Tregs) and Myeloid derived suppressor cells (MDSCs) in tumor tissues by multicolor flow cytometry Dot plots of representative patient are displayed. Multiple antibodies were used to sort out the appropriate immune cell lineages among tumor cells. A. Tregs were defined as CD45+CD4+CD25± and FOXP3+. B. MDSCs were defined as CD45+, CD11b+, HLA−DR− and CD14+.

We defined helper T cells as CD45+ CD3+ CD4+, cytotoxic T cells as CD45+ CD3+ CD8+, Tregs as CD45+ CD4+ CD25± and FOXP3+, and MDSCs as CD45+ CD11b+ HLA−DR− and CD14+ [21, 22, 27, 44–48] (Figure 3). The frequency of each cell subgroup among the total examined cells was determined. We also assessed the percentages of the following immune cell subsets among leukocytes: CD8+ T cells among CD3+ T cells, Tregs among CD4+ T cells, and MDSCs among total CD45+ leukocytes. To identify optimal cutoffs with the highest statistical significance for patient survival, we used X-Tile statistical software (Yale University, New Haven, CT, USA) [49] and grouped the cases into low- and high-density groups for each marker following dichotomization.

### Statistics

Pearson's chi-square test, Fisher's exact test, and the Cochran-Armitage test were used as appropriate for statistical analysis. Disease-free survival (DFS) was defined as the time from surgery to the first relapse of cancer, occurrence of a second primary tumor, or death from any cause. Overall survival (OS) was measured from the date of surgery to the date of death. The OS and DFS were calculated using the Kaplan-Meier method, and survival curves were compared by the log-rank method. The Cox proportional hazard model was used to evaluate the association between clinicopathologic factors and survival for multivariate analysis. In the multivariate analysis, variables showing *p* values of less than 0.3 in the univariate analysis were included as covariates. All statistical analyses were performed using SPSS software (SPSS Inc., Chicago, IL, USA) or R software (version 3.03), and *p* values less than 0.05 were considered to be statistically significant.
